# Identification of co-expressed genes associated with *MLL* rearrangement in pediatric acute lymphoblastic leukemia

**DOI:** 10.1042/BSR20200514

**Published:** 2020-05-07

**Authors:** Hao Zhang, Bei Liu, Juan Cheng, Haizhen Ma, Zijian Li, Yaming Xi

**Affiliations:** Department of Hematology, The First Hospital of Lanzhou University, Lanzhou, Gansu Province, China

**Keywords:** acute lymphoblastic leukemia, MLL rearrangement, pediatric, WGCNA

## Abstract

Rearrangements involving the mixed lineage leukemia (*MLL*) gene are common adverse prognostic factors of pediatric acute lymphoblastic leukemia (ALL). Even allogeneic hematopoietic stem cell transplantation does not improve the outcome of ALL cases with some types of *MLL* rearrangements. The aim of the present study was to identify the co-expressed genes that related to *MLL* rearrangement (*MLL*-r) and elucidate the potential mechanisms of how *MLL*-r and their partner genes lead to leukemogenesis. Gene co-expression networks were constructed using the gene expression data and sample traits of 204 pretreated pediatric ALL patients, and co-expression modules significantly related to the *MLL*-r were screened out. Gene ontology annotation and Kyoto Encyclopedia of Genes and Genomes pathway analysis of the module genes were performed. Hub genes were identified and their expression levels were analyzed in samples with or without *MLL*-r and the results were validated by an independent investigation. Furthermore, the relationships between the hub genes and sample traits were analyzed. In total, 21 co-expression modules were identified. The green module was positively correlated with *MLL*-r. *PROM1, LGALS1, CD44, FUT4* and *HOXA10* were identified as hub genes, which were involved in focal adhesion, calcium-dependent phospholipid binding, connective tissue development and transcriptional misregulation in cancer. The expression levels of the five hub genes were significantly increased in *MLL*-r samples, and the results were further validated. *PROM1, LGALS1, CD44* and *HOXA10* were positively related to the leukocyte count. These findings might provide novel insight regarding the mechanisms and potential therapeutic targets for pediatric ALL with *MLL*-r.

## Introduction

Acute lymphoblastic leukemia (ALL) is the most common cancer diagnosed in children and represents approximately 25% of cancer diagnoses among children [1]. Approximately 98% of patients with pediatric ALL achieve remission, and 85% of patients aged 1–18 years with newly diagnosed ALL treated on current regimens are expected to be long-term event-free survivors, with over 90% surviving at 5 years [2,3]. Unfortunately, despite advances in treatments according to risk stratification, ALL remains the most common cause of death due to malignancy in children [4,5]. Several high-risk cytogenetic and molecular subtypes of ALL are associated with unfavorable outcomes. Rearrangements involving the mixed lineage leukemia (*MLL*) gene (also known as the *KMT2A* gene), which occur in approximately 5–10% of overall childhood ALL cases, are common adverse prognostic factors of ALL [6,7]. *MLL* gene rearrangement (*MLL*-r) is significantly associated with high risks of treatment failure, relapse and CNS involvement [8]. The 5-year event-free survival (EFS) and overall survival (OS) rates of these patients are worse than those of patients without *MLL* rearrangement (non-*MLL*-r). MLL-AF4 or MLL-AF9 is associated with poorer outcome (5-year EFS<60%) than other types of *MLL*-r [9]. Even worse, allogeneic hematopoietic stem cell transplantation (allo-HSCT) with HLA-matched related or unrelated donors has failed to improve the outcome of ALL cases with MLL-AF4 [7,9,10]. The *MLL* gene codes for large nuclear protein histone lysine methyltransferase 2A (KMT2A) functions as a transcriptional regulator that catalyzes the methylation of histone H3 lysine 4 (H3K4). It has been confirmed that *MLL* participates in the regulation of hematopoietic differentiation, while *MLL*-r leads to hematological abnormalities, further contributing to leukemogenesis [11,12]. However, MLL fusion proteins are unable to promote leukemogenesis on their own [13]; some additional events such as high levels of *MEIS1* and *HOX* gene expression [7,14]; and *FLT3* activation [15], *K-Ras* mutations [16] and epigenetic abnormalities [17] are required. Not only that, these cooperative events have only been found in a portion of leukemia cases with *MLL*-r, indicating that these abnormities might not be essential events for the *MLL*-dependent leukemogenic process. Thus far, the exact mechanisms of how *MLL*-r causes leukemogenesis remain unclear.

Because traditional approaches, including chemotherapy and allo-HSCT, are curative for few patients with pediatric ALL and *MLL*-r. Several novel targeted treatment options are emerging. FLT-3 inhibitors, including lestaurtinib [18] and quizartinib [19], have been used in clinical trials of patients with leukemia and *MLL*-r. The results failed to demonstrate that FLT-3 inhibitors are a beneficial therapeutic approach. The proteasome inhibitor bortezomib has been used alone in five leukemia patients with *MLL*-r, while only three cases showed temporary hematologic responses and the other two cases showed no response. Pinometostat, which is an inhibitor of the H3K79 methyltransferase DOT1L, has shown promising efficacy in a preclinical study of *MLL*-r leukemia [20,21]. However, the result was disappointing when it was used in patients with pediatric ALL and *MLL*-r [22]. Another epigenetic agent, histone deacetylase (HDAC) inhibitor, has been used in a clinical trial for the treatment of *MLL*-r acute leukemias in pediatric patients, but its efficacy has yet to be demonstrated. Immunotherapy, particularly chimeric antigen receptor (CAR) T-cell technology, is a promising therapeutic regimen for high-risk B-ALL. However, the application is limited due to the low/negative expression levels of targeted antigens, such as CD19 and CD22, in ALL cases with *MLL*-r [23–25]. In a word, only a minority of pediatric ALL patients with *MLL*-r may benefit from these directed strategies. Thus, it is urgently necessary to identify innovative treatment approaches to improve the unfavorable prognosis of these patients.

In the present study, a co-expression network was constructed by weighted gene co-expression network analysis (WGCNA) to identify co-expressed gene modules. The aim of the present study is to investigate the genes significantly related to *MLL*-r ALL and elucidate the mechanisms of how MLL fusions and their partner genes lead to leukemogenesis, which may provide novel therapeutic targets for ALL with *MLL*-r.

## Materials and methods

### Gene expression data and sample trait collection

The dataset GSE68735, containing microarray data and clinical phenotype information from 207 pretreated patients with pediatric ALL, was downloaded from the GEO database (https://www.ncbi.nlm.nih.gov/geo/). The gene expression data were generated by an Affymetrix Human Genome U133 Plus 2.0 Array. The sample traits, including the age, sex, leukocyte count, *MLL*-r, *TEL-AML, E2A-PBX1*, combined trisomy 4 and 10 and central nervous system (CNS) statuses, of 207 ALL cases were extracted.

### Co-expression network construction and module identification

Scale-free gene co-expression networks were constructed using the WGCNA package in R [26]. Outlier samples were identified and removed before co-expression analysis. A similarity matrix of all pairwise genes was constructed based on Pearson’s correlation analysis. The soft-thresholding power β was used when the scale-free topology fit index was approximately 0.90, which caused the matrix to achieve a scale-free network. The similarity matrix was converted into an adjacency matrix, and the adjacency matrix was then transformed into a topological overlap matrix (TOM). The dynamic tree cut method was used to identify co-expressed gene modules with a minimum gene group size of 30. The module eigengene (ME) was used to represent the expression profiles of the module genes [27]. Similar modules with correlations > 0.75 were merged.

### Module–trait relationship analysis

Relationships between the modules and sample traits were identified by calculating the correlation coefficient. In the present study, the *MLL*-r status was selected as a target sample trait. Co-expression modules significantly related to the *MLL*-r status were screened out for the subsequent analysis. The correlation between a specific gene and the eigengene of a module (module membership, MM) and the correlation between a specific gene and sample traits (gene significance, GS) were calculated in the candidate modules.

### Gene functional annotation and pathway enrichment analysis

Gene ontology (GO) functional annotation and Kyoto Encyclopedia of Genes and Genomes (KEGG) pathway enrichment analysis of the module genes mentioned above were performed with the clusterProfiler package in R [28] with an adjusted threshold of *P* < 0.05.

### Hub gene identification and validation

Genes with high connectivity in the network were identified by their kME (eigengene connectivity) values. Genes with |kME| ≥ 0.8 were identified as candidate hub genes. A protein–protein interaction (PPI) network of the module genes was constructed using the STRING online database (version 10.5, http://www.string-db.org/) [29], with a minimum required interaction score > 0.4 (median confidence). Hub genes were screened out by the CytoHubba plug-in (version 2.1.6, http://apps.cytoscape.org/apps/cytohubba) in Cytoscape [30]. The same hub genes identified by the two methods were selected and defined as hub genes. The hub gene mRNA expression in samples with or without *MLL*-r was analyzed, and the results were validated by the Coustan-Smith leukemia study [31] in the Oncomine database (https://www.oncomine.org/).

### The relationship between hub genes and sample traits

To reveal the relationship between the hub genes and sample traits, correlation analysis was performed using Spearman’s correlation method.

## Results

### Gene expression data and sample traits

The dataset GSE68735 contained expression values for 207 patients diagnosed with pediatric ALL. Three cases, which included GSM1679955, GSM1680005 and GSM1680015, were excluded because the *TEL-AML* and trisomy 4 and 10 statuses were unknown. A total of 204 cases with a median age of 13 years were selected for further analysis. *MLL*-r was identified in approximately 10% of the patients. Three of the 207 cases had *TEL-AML*, 23 cases had *E2A-PBX1*, and 5 cases had combined trisomy 4 and 10. The clinical and laboratory characteristics of patients according to the *MLL* rearrangement status are shown in Table 1.

**Table 1:** Clinical and laboratory characteristics of patients according to *MLL* rearrangement status

Characteristics	*MLL*-r (*n* = 21)	non-*MLL*-r (*n* = 183)	*P*-value
Age (days)	1704.0 (681.0–4950.5)	4918.0 (1914.0–5867.0)	0.005*
Gender (*n*)			
Male	11	123	0.175^#^
Female	10	60	
Leukocyte count (×10^9^/l)	125.8 (44.3–241.7)	45.1 (12.7–137.8)	0.011*
CNSL (*n*)			
No	20	163	0.378^#^
Yes	1	20	

* The difference of age and Leukocyte count between *MLL*-r and non-*MLL*-r group was compared by Mann–Whitney *U* test.

# The difference of gender and CNSL between *MLL*-r and non-*MLL*-r group was compared by chi-square test. CNSL: central nervous system leukemia; *MLL-*r: mixed lineage leukemia gene rearrangement; Non-*MLL-*r: without mixed lineage leukemia gene rearrangement.

### Co-expression network construction and module identification

**Figure 1:**
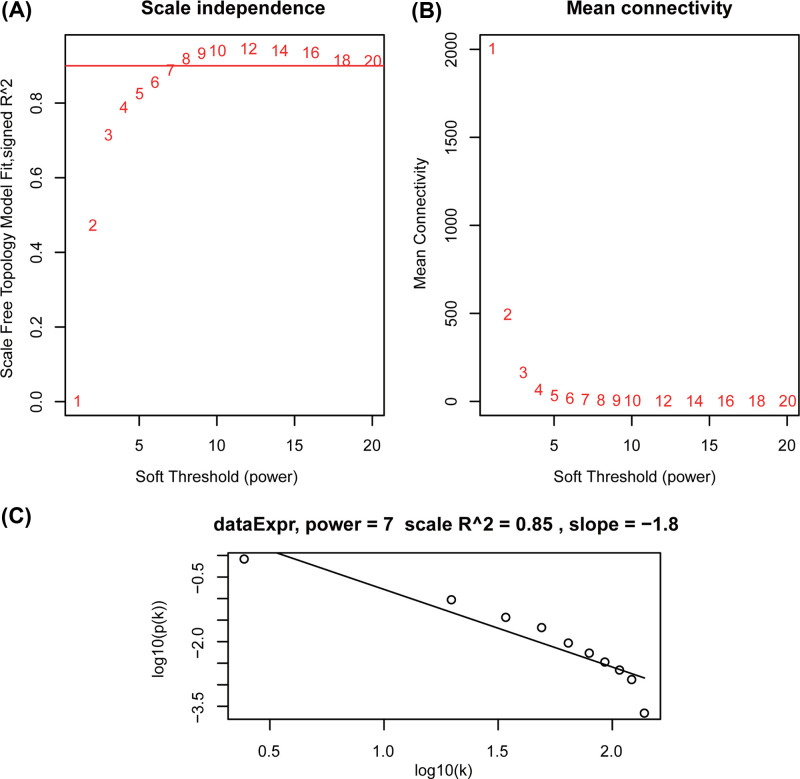
Determination of soft-thresholding power β (**A**) The scale-free topology fit index for various value of β. (**B**) The mean connectivity for various value of β. (**C**) Checking the scale-free network when β = 7.

**Figure 2:**
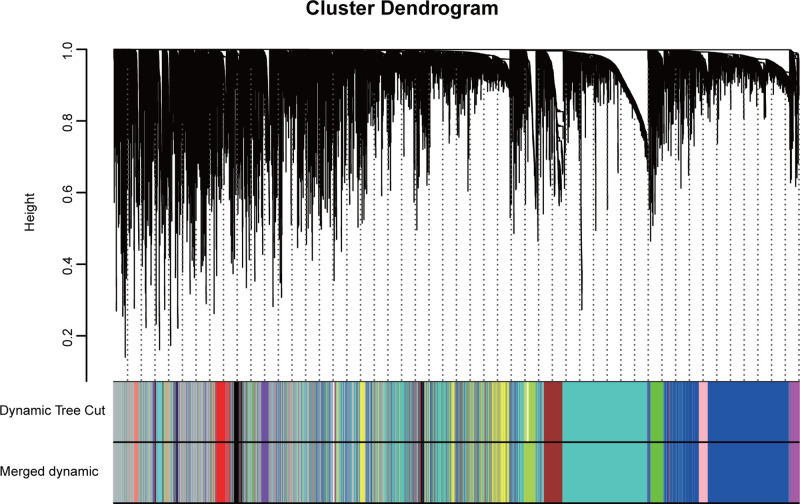
Gene clustering dendrogram based on dissimilarity measure Co-expression modules were identified by the dynamic tree cut method and similar modules were merged when the correlation higher than 0.75. Each co-expression module was assigned a distinctive color, and each vertical line represents a gene.

Two outlier samples were removed based on the sample clustering result. The top 25% (13669) of the most variant genes were selected to construct the co-expression network. The scale-free topology fit index reached 0.85 when β was set to 7 (Figure 1). As a result, 21 different co-expression modules clustered from 37 to 3654 genes were identified (Figure 2).

### Module–trait relationship analysis

**Figure 3:**
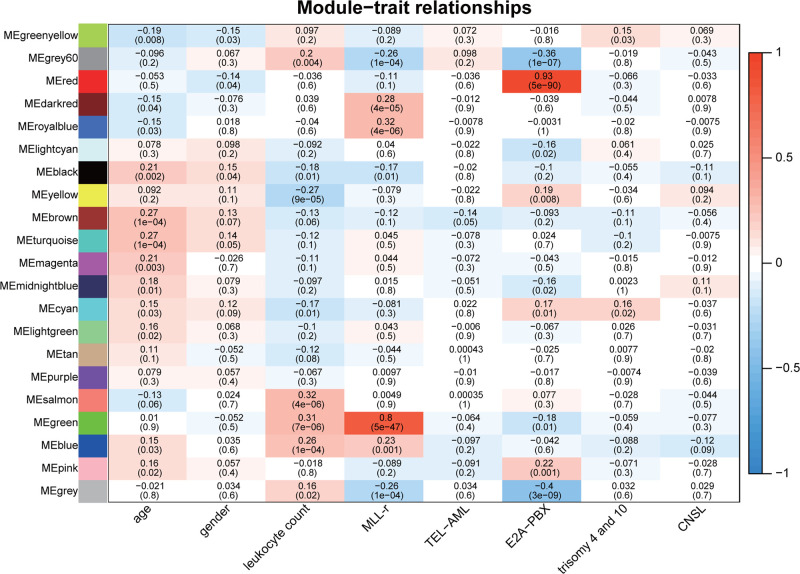
Correlation between co-expression modules and sample traits Each row represents a module eigengene and each column represents a sample trait. Each cell contains a correlation (the first line) and *P*-value (the second line). The cells are color-coded by correlation according to the color legend. CNSL: central nervous system leukemia; *MLL*: mixed lineage leukemia.

**Figure 4:**
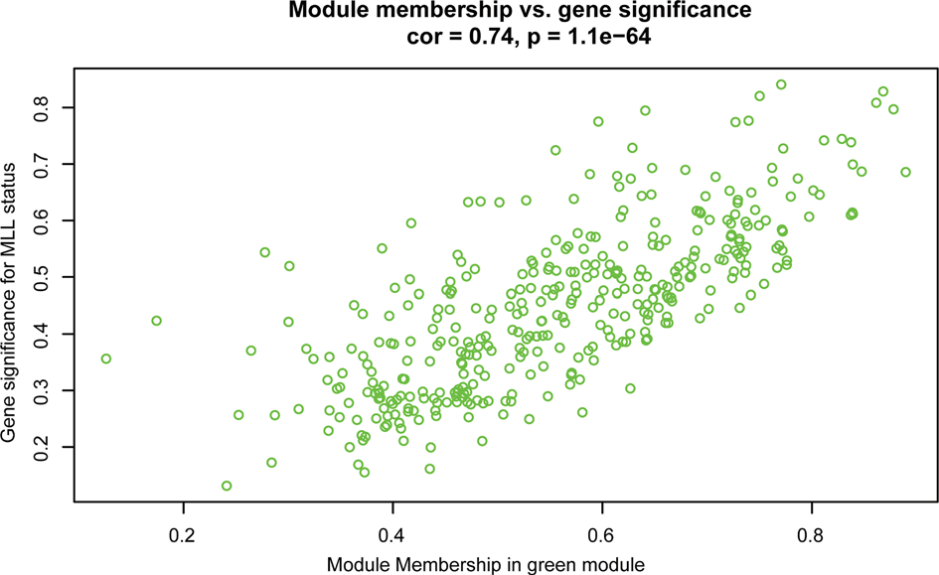
Scatter plot of GS for *MLL* gene rearrangement status versus MM in green module GS: gene significance; *MLL*: mixed lineage leukemia; MM: module membership.

The green module was positively related to the *MLL*-r status (*r* = 0.8, *P* = 5e-47) and leukocyte count (*r* = 0.31, *P* = 7e-06) (Figure 3). Moreover, the GS for the *MLL*-r status and the MM of genes in the green module were calculated, and a scatter plot of the correlation was generated. The result showed that there was a significantly positive correlation between the GS for the *MLL*-r status and the MM of genes in the green module (Figure 4).

### GO annotation and pathway enrichment analysis of the genes in the green module

**Figure 5:**
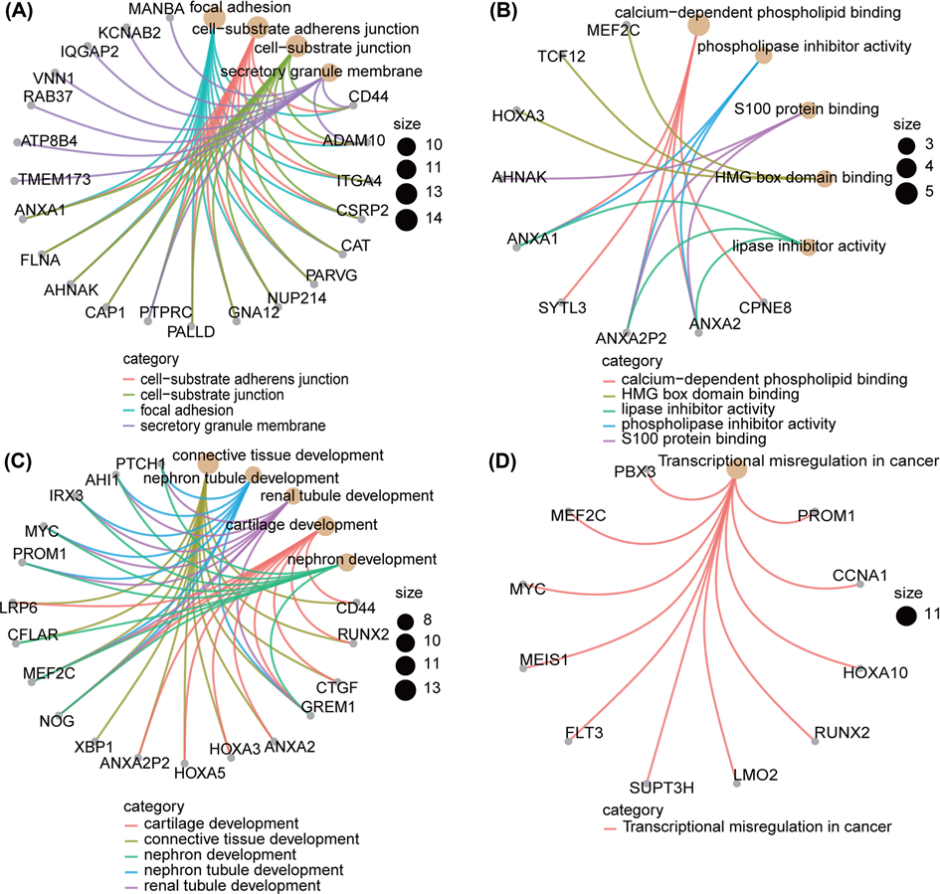
GO functional annotation and KEGG pathway enrichment of genes in green module Each orange circle represents a term and gray circle represents a gene. (**A**) GO-CC terms. (**B**) GO-MF terms. (**C**) GO-BP terms. (**D**) KEGG pathway. BP: biological processes; CC: cellular component; KEGG: Kyoto Encyclopedia of Genes and Genomes; MF: molecular function.

All 213 genes in the green module were analyzed by the clusterProfiler package in R for GO annotation and KEGG analysis. The results revealed that most significant terms for cellular component (CC), molecular function (MF) and biological processes (BPs) were focal adhesion (GO:0005925), calcium-dependent phospholipid binding (GO:0005544) and connective tissue development (GO:0061448), respectively (Figure 5A–C). The KEGG analysis indicated that 11 genes in the green module were significantly enriched in transcriptional misregulation in cancer (hsa05202) (Figure 5D).

### Hub gene identification and validation

**Figure 6:**
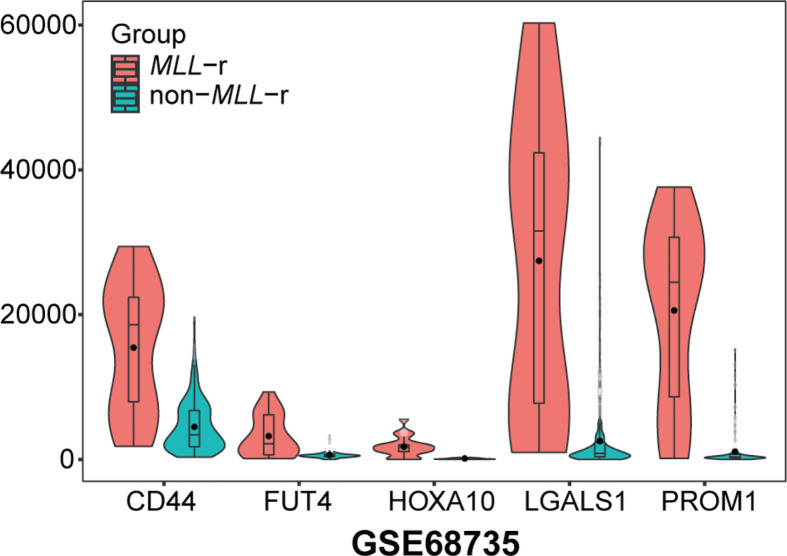
Analysis of the mRNA expression value of five hub genes in pediatric ALL patients with or without *MLL* rearrangement *P* values are the results according to Mann–Whitney *U* test. *MLL-*r: mixed lineage leukemia gene rearrangement; non-*MLL-*r: without mixed lineage leukemia gene rearrangement.

**Figure 7:**
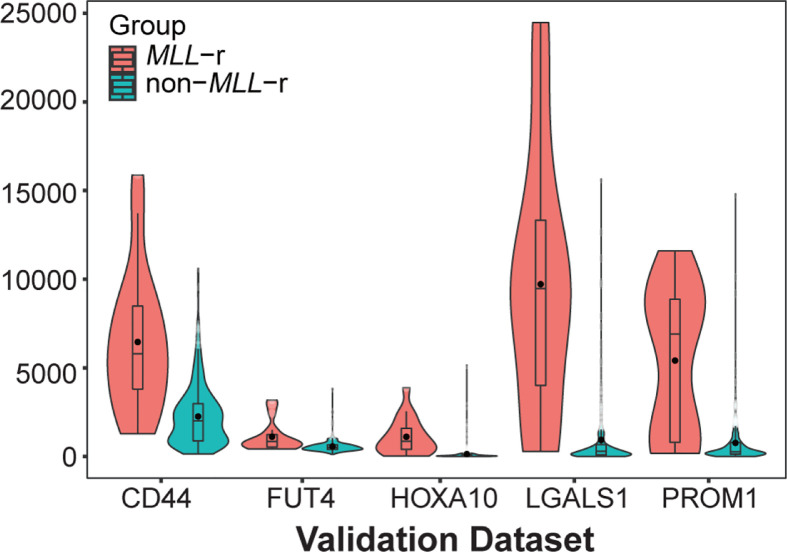
Validation of the hub genes’ expression level in the Oncomine database *P* values are the results according to Mann–Whitney *U* test. *MLL-*r: mixed lineage leukemia gene rearrangement; non-*MLL-*r: without mixed lineage leukemia gene rearrangement

*PROM1* (ENSG00000007062; chr4:15,963,076-16,084,378), *LGALS1* (ENSG00000100097; chr22:37,675,636-37,679,802), *CD44* (ENSG00000026508; chr11:35,138,870-35,232,402), *FUT4* (ENSG00000196371; chr11:94,543,840-94,549,898) and *HOXA10* (ENSG00000253293; chr7:27,170,592-27,180,261) with kME values > 0.8 were simultaneously identified as hub genes by the CytoHubba plug-in in Cytoscape. The expression levels of all five hub genes were significantly increased in the samples with *MLL*-r compared with the samples without *MLL*-r (Figure 6). Only one study that compared the expression levels of the five hub genes in patients with pediatric ALL with or without *MLL*-r was identified in the Oncomine database. Similarly, all five hub genes showed significantly higher expression levels in patients with pediatric ALL and *MLL*-r (Figure 7).

### The relationship between hub genes and sample traits

**Figure 8:**
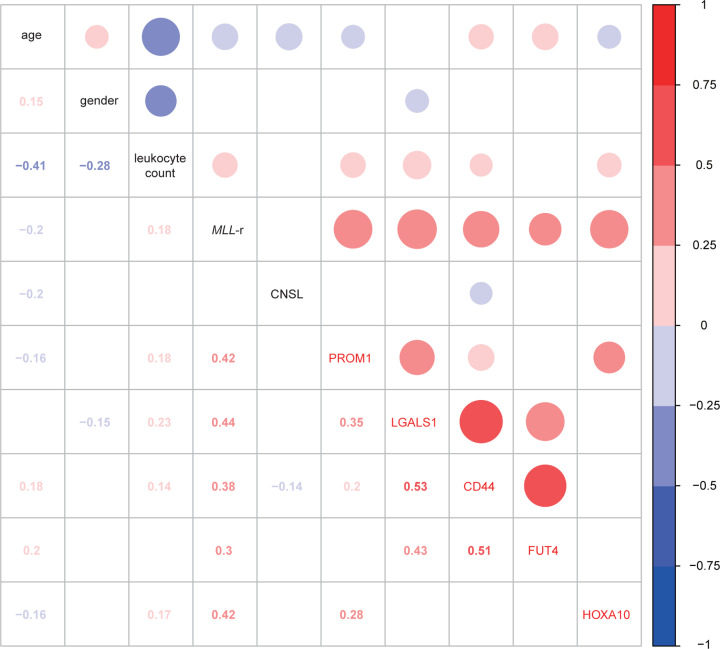
Heatmap of relationship between hub genes and sample traits Circles in the cells upper right represent correlation of hub genes and sample traits; red represents positive correlation; blue represents negative correlation; the gradual color change from blue to red represents the correlation coefficients change from low to high; the size of circles represents size of correlation coefficients. Numbers in the cells lower left represent the correlation *P*-values. Insignificant correlations with *P*-value lower than 0.05 are leaved blank. The correlation coefficients were calculated using Spearman’s correlation method; CNSL: central nervous system leukemia; *MLL*: mixed lineage leukemia.

As mentioned above, the green module was positively correlated with the *MLL*-r status and leukocyte count. The results of the correlation analysis revealed that *PROM1, LGALS1, CD44* and *HOXA10* were positively correlated with the leukocyte count with *P*-values less than 0.05 (Figure 8).

## Discussion

*MLL*-r remains a major unfavorable prognostic factor in ALL. The EFS and OS of pediatric ALL with *MLL*-r are inferior to those of patients without *MLL*-r due to chemotherapeutic refractoriness and relapse. It remains challenging to elucidate the molecular mechanisms of *MLL*-r leukemia and identify new therapeutic targets. Therefore, WGCNA was carried out to identify co-expressed genes associated with *MLL*-r in pediatric ALL. The green module was screened for further analysis because it had the highest correlation coefficient with the *MLL*-r status of pediatric ALL. To understand the biological functions of the co-expressed gene module, GO annotation and pathway enrichment analysis were performed. The results showed that the most significant GO-CC term enriched by the co-expressed genes was focal adhesion, which is a crucial step in cell migration [32]. The calcium-dependent phospholipid binding (MF term) and connective tissue development (BP term) were mainly involved in the green module genes. The most significantly enriched KEGG pathway in the green module was transcriptional misregulation in cancer, which is involved in ALL with *MLL*-r.

To distinguish important genes in the green module, a PPI network of the module genes was constructed. Then, hub genes, which included *PROM1, LGALS1, CD44, FUT4* and *HOXA10*, were identified by CytoHubba based on the PPI network, and all hub genes had kME values ≥ 0.8. The expression levels of all five hub genes were significantly increased in the samples with *MLL*-r compared with the samples without *MLL*-r, and the results were validated by an independent investigation in the Oncomine database. Furthermore, the results of the correlation analysis revealed that *PROM1, LGALS1, CD44* and *HOXA10* were positively correlated with the leukocyte count, which is an adverse prognostic factor of pediatric ALL. The *HOXA10* gene, which is a member of the homeobox gene family, is typically expressed in decidualizing stromal cells and hematopoietic stem cells. It codes a transcription factor that participates in hematopoietic cell differentiation and normal implantation [33–35]. The abnormal expression of homeoboxgenes, which contributed to oncogenesis and progression of cancers, were found in breast, colon and ovarian cancers [36]. *HOXA10, HOXA9* and *HOXC6* were consistently up-regulated in cell lines and primary cells from ALL patients with *MLL*-r [14,37]. Importantly, *HOX* genes, including *HOXA10, HOXA9* and *HOXC6*, are significantly up-regulated in both T- and B-lineage ALLs with *MLL* translocations. Conversely, homeobox genes knockdown decreased the proliferation of leukemia cells of acute leukemia with MLL-r [38]. These results strongly indicated that these genes are involved in *MLL*-r ALL as central factors. The possible mechanism could be that the dysregulation of *HOX* genes directly caused by MLL fusion proteins promotes the excessive self-renewal of hematopoietic cells, which is associated with the origin and maintenance of leukemogenic events [39].

*CD44*, which encodes a cell adhesion glycoprotein, is crucial to various physiological processes, such as cell migration, limb development, extracellular binding, lymphocyte homing and hematopoiesis. Additionally, *CD44* plays crucial roles in pathological processes, particularly in tumor progression, metastasis and chemoresistance, suggesting that it is a potential marker of stem cells in solid tumors and leukemia-initiating cells. At present, CD44 was identified as a stemness-related gene of several cancers including glioblastoma, breast cancer, hepatocellular carcinoma, colorectal cancer and acute myeloid leukemia [40]. Significantly higher expression levels were observed in patients with ALL compared with umbilical cord blood precursor cells [41] and contributed to chemoresistance by increasing drug efflux [42]. Interestingly, the gene expression level of *CD44* in ALL cases with *MLL*-r was much greater than that in ALL cases without *MLL*-r [43,44], which was consistent with the results of our study. These findings demonstrated that *MLL*-r may participate in the maturation arrest of early lymphoid progenitors because *CD44* expression is involved in early steps of lymphoid development [45]. Previous studies have shown that anti-CD44 monoclonal antibodies inhibit the proliferation and reverse the differentiation arrest of acute myeloid leukemia cell lines and primary acute myeloid leukemia cells by increasing the expression level and stability of the p27 protein [46–48]. Moreover, anti-CD44 monoclonal antibodies have also shown the promising potential of eradicating acute myeloid leukemia stem cells by inhibiting acute myeloid leukemia stem cell homing [49]. Whether CD44-targeted therapy is an effective strategy for the treatment of ALL with *MLL*-r and acute myeloid leukemia requires further investigation. The *PROM1* gene encodes the surface antigen CD133, which acts as a marker of hematopoietic stem cells and progenitor cells [50]. In addition, CD133 was used for identifying many types of cancer stem cells (CSCs) [40]. And CD133+ cells were characterized by self-renewal, proliferation, invasion and multi-drug resistance, the mechanism was associated with activation of the CSCs-related signaling such as PI3K/Akt, epidermal growth factor receptor and src-focal adhesion kinase [51]. Overexpression of the *PROM1* gene has been identified in ALL with MLL-AF4 [37]. *PROM1* transcription, which is required for the growth of ALL cells and is regulated by AF4, is downregulated due to the knockdown of AF4 [52]. However, few studies have focused on the expression level and effect of *PROM1* in ALL with other MLL translocations. Recently, a bispecific chimeric antigen receptor (CAR) targeting CD19 and CD133 was designed, and tandem CAR (TanCAR) T cells were then applied to cell lines and a mouse model with MLL-AF4 B-cell ALL, which is frequently accompanied by the loss of CD19 expression. The results showed that TanCAR T cells selectively kill CD19+CD133+ and CD19-CD133+ leukemia cells with slight cytotoxicity against normal hematopoietic stem cells and progenitor cells [53]. But other investigators have highlighted concerns regarding CAR targeting CD133 as a suitable approach for treating *MLL*-r leukemia because their data indicated that the expression level of PROM1/CD133 in early normal hematopoietic stem cells is equal to or greater than that in B-cell ALL with MLL-AF4 or MLL-AF9 [54]. Thus, the potential of PROM1/CD133 as a target for immunotherapy requires further confirmation. The *LGALS1* gene encodes galectin-1 (Gal-1), which is acarbohydrate-binding protein with the ability to bind β-D-galactopyranoside glycans and is involved in glycosylation [55,56]. The ligation of galectin and glycan contributes to cell proliferation, apoptosis, migration and tumor immunity [56]. However, the dysregulation of glycosylation and galectin expression have been shown to be significant events in oncogenesis, metastasis and resistance to chemotherapy [55,57,58]. The gene and protein expression levels of *LGALS1* have been shown to be significantly higher in MLL-r B-ALL due to MLL-dependent epigenetic modifications [59]. Several inhibitors designed to target galectin-1 have shown therapeutic potential in different tumors and inflammatory disorders [60]. PTX008, which is an inhibitor of Gal-1, have been shown to reduce the proliferation and migration of B-cell precursor cells in ALL [61]. However, the challenge is that cancer cells produce different galectin isoforms by alternative splicing, which may result in resistance to galectin inhibitors [62]. Coincidentally, the fucosyltransferase-4 (*FUT4*) gene encode sanα-1,3-fucosyl transferase named Fut-IV and participates in fucosylation, which is an important type of glycosylation. Aberrant fucosylation has been shown in a number of pathological events, including the proliferation, invasion and metastasis of cancer cells and chemoresistance [63,64]. FucT-IV is distributed in leukocytes and myeloid cells and contributes to the final step of Lewis (Le) antigen biosynthesis [65]. The high expression levels of FucTs accompanied by the overexpression of certain glycan structures have been identified in several cancers. Similarly, increased expression levels of Le^x^ have been observed in various solid tumors [66]. To date, few studies have examined the relationship between the *FUT4* gene and leukemia, and most of these studies focused on myeloid leukemia cell lines [67,68]. Recently, a study revealed that *FUT4* is overexpressed in patients with ALL [69]. However, the relationship between the *FUT4* gene and *MLL*-r status remains unknown and requires further exploration.

In conclusion, a scale-free co-expression network was constructed by WGCNA. The green module was identified as a meaningful module that is significantly related to *MLL*-r, and *PROM1, LGALS1, CD44, FUT4* and *HOXA10* in this module were identified as hub genes with higher mRNA expression levels in *MLL*-r cases than in non-*MLL*-r cases. Additionally, the results were validated in other independent database. Interestingly, among these hub genes, *HOXA10, CD44* and *PROM1* were all recognized as important markers of hematopoietic stem cells and progenitor cells. Both *LGALS1* and *FUT4* are involved in the dysregulation of glycosylation. Their overexpression may play dominant roles in the leukemogenesis, development, chemoresistance and relapse of *MLL*-r ALL by promoting the characteristic self-renewal of leukemia stem cells. Overall, these findings might provide novel insights regarding the mechanisms and potential therapeutic targets for patients with pediatric ALL and *MLL*-r.

## Abbreviations

ALL: acute lymphoblastic leukemia
allo-HSCT: allogeneic hematopoietic stem cell transplantation
CAR: chimeric antigen receptor
CSC: cancer stem cell
EFS: event-free survival
*FUT4*: fucosyltransferase-4
GO: gene ontology
H3K4: histone H3 lysine 4
HDAC: histone deacetylase
KEGG: Kyoto Encyclopedia of Genes and Genomes
KMT2A: histone lysine methyltransferase 2A
*MLL*: mixed lineage leukemia
*MLL*-r: *MLL* rearrangement
OS: overall survival
PPI: protein–protein interaction
WGCNA: weighted gene co-expression network analysis

## References

[1] Pui C.H. and Evans W.E. (2013) A 50-year journey to cure childhood acute lymphoblastic leukemia. Semin. Hematol. 50, 185–196 10.1053/j.seminhematol.2013.06.007 23953334 PMC3771494

[2] Moricke A., Zimmermann M., Valsecchi M.G., Stanulla M., Biondi A., Mann G. et al. (2016) Dexamethasone vs prednisone in induction treatment of pediatric ALL: results of the randomized trial AIEOP-BFM ALL 2000. Blood 127, 2101–2112 10.1182/blood-2015-09-670729 26888258

[3] Pieters R., de Groot-Kruseman H., Van der Velden V., Fiocco M., van den Berg H., de Bont E. et al. (2016) Successful Therapy Reduction and Intensification for Childhood Acute Lymphoblastic Leukemia Based on Minimal Residual Disease Monitoring: Study ALL10 From the Dutch Childhood Oncology Group. J. Clin. Oncol. 34, 2591–2601 10.1200/JCO.2015.64.6364 27269950

[4] Chessells J.M., Veys P., Kempski H., Henley P., Leiper A., Webb D. et al. (2003) Long-term follow-up of relapsed childhood acute lymphoblastic leukaemia. Br. J. Haematol. 123, 396–405 10.1046/j.1365-2141.2003.04584.x 14616997

[5] Gaynon P.S. and Sun W. (2016) Oligoclonality and new agent evaluation in acute lymphoblastic leukaemia. Br. J. Haematol. 173, 950–957 10.1111/bjh.14143 27221005

[6] Behm F.G., Raimondi S.C., Frestedt J.L., Liu Q., Crist W.M., Downing J.R. et al. (1996) Rearrangement of the MLL gene confers a poor prognosis in childhood acute lymphoblastic leukemia, regardless of presenting age. Blood 87, 2870–2877 10.1182/blood.V87.7.2870.bloodjournal8772870 8639906

[7] Winters A.C. and Bernt K.M. (2017) MLL-Rearranged Leukemias-An Update on Science and Clinical Approaches. Front. Pediatr. 5, 4 10.3389/fped.2017.00004 28232907 PMC5299633

[8] Pui C.H. (2000) Acute lymphoblastic leukemia in children. Curr. Opin. Oncol. 12, 3–12 10.1097/00001622-200001000-00002 10687723

[9] Pui C.H., Gaynon P.S., Boyett J.M., Chessells J.M., Baruchel A., Kamps W. et al. (2002) Outcome of treatment in childhood acute lymphoblastic leukaemia with rearrangements of the 11q23 chromosomal region. Lancet 359, 1909–1915 10.1016/S0140-6736(02)08782-2 12057554

[10] Pui C.H., Chessells J.M., Camitta B., Baruchel A., Biondi A., Boyett J.M. et al. (2003) Clinical heterogeneity in childhood acute lymphoblastic leukemia with 11q23 rearrangements. Leukemia 17, 700–706 10.1038/sj.leu.2402883 12682627

[11] Guenther M.G., Jenner R.G., Chevalier B., Nakamura T., Croce C.M., Canaani E. et al. (2005) Global and Hox-specific roles for the MLL1 methyltransferase. Proc. Natl. Acad. Sci. U.S.A. 102, 8603–8608 10.1073/pnas.0503072102 15941828 PMC1150839

[12] Yagi H., Deguchi K., Aono A., Tani Y., Kishimoto T. and Komori T. (1998) Growth disturbance in fetal liver hematopoiesis of Mll-mutant mice. Blood 92, 108–117 10.1182/blood.V92.1.108.413k11_108_117 9639506

[13] Stam R.W. (2013) The ongoing conundrum of MLL-AF4 driven leukemogenesis. Blood 121, 3780–3781 10.1182/blood-2013-03-491738 23660854

[14] Armstrong S.A., Golub T.R. and Korsmeyer S.J. (2003) MLL-rearranged leukemias: insights from gene expression profiling. Semin. Hematol. 40, 268–273 10.1016/S0037-1963(03)00196-3 14582077

[15] Bueno C., Ayllon V., Montes R., Navarro-Montero O., Ramos-Mejia V., Real P.J. et al. (2013) FLT3 activation cooperates with MLL-AF4 fusion protein to abrogate the hematopoietic specification of human ESCs. Blood 121, 3867–3878, S3861-S3863 10.1182/blood-2012-11-470146 23479570

[16] Tamai H., Miyake K., Takatori M., Miyake N., Yamaguchi H., Dan K. et al. (2011) Activated K-Ras protein accelerates human MLL/AF4-induced leukemo-lymphomogenicity in a transgenic mouse model. Leukemia 25, 888–891 10.1038/leu.2011.15 21311557

[17] Guest E.M. and Stam R.W. (2017) Updates in the biology and therapy for infant acute lymphoblastic leukemia. Curr. Opin. Pediatr. 29, 20–26 10.1097/MOP.0000000000000437 27841777

[18] Brown P., Levis M., Shurtleff S., Campana D., Downing J. and Small D. (2005) FLT3 inhibition selectively kills childhood acute lymphoblastic leukemia cells with high levels of FLT3 expression. Blood 105, 812–820 10.1182/blood-2004-06-2498 15374878

[19] Cooper T.M., Cassar J., Eckroth E., Malvar J., Sposto R., Gaynon P. et al. (2016) A Phase I Study of Quizartinib Combined with Chemotherapy in Relapsed Childhood Leukemia: A Therapeutic Advances in Childhood Leukemia & Lymphoma (TACL) Study. Clin. Cancer Res. 22, 4014–4022 26920889 10.1158/1078-0432.CCR-15-1998

[20] Daigle S.R., Olhava E.J., Therkelsen C.A., Basavapathruni A., Jin L., Boriack-Sjodin P.A. et al. (2013) Potent inhibition of DOT1L as treatment of MLL-fusion leukemia. Blood 122, 1017–1025 10.1182/blood-2013-04-497644 23801631 PMC3739029

[21] Daigle S.R., Olhava E.J., Therkelsen C.A., Majer C.R., Sneeringer C.J., Song J. et al. (2011) Selective killing of mixed lineage leukemia cells by a potent small-molecule DOT1L inhibitor. Cancer Cell 20, 53–65 10.1016/j.ccr.2011.06.009 21741596 PMC4046888

[22] Shukla N., O'Brien M.M., Silverman L.B., Pauly M., Wetmore C., Loh M.L. et al. (2015) Preliminary Report of the Phase 1 Study of the DOT1L Inhibitor, Pinometostat, EPZ-5676, in Children with Relapsed or Refractory MLL-r Acute Leukemia: Safety, Exposure and Target Inhibition. Blood 126, 3792–3792 10.1182/blood.V126.23.3792.3792

[23] Aoki Y., Watanabe T., Saito Y., Kuroki Y., Hijikata A., Takagi M. et al. (2015) Identification of CD34+ and CD34- leukemia-initiating cells in MLL-rearranged human acute lymphoblastic leukemia. Blood 125, 967–980 10.1182/blood-2014-03-563304 25538041 PMC4319237

[24] Gardner R., Wu D., Cherian S., Fang M., Hanafi L.A., Finney O. et al. (2016) Acquisition of a CD19-negative myeloid phenotype allows immune escape of MLL-rearranged B-ALL from CD19 CAR-T-cell therapy. Blood 127, 2406–2410 10.1182/blood-2015-08-665547 26907630 PMC4874221

[25] Shah N.N., Stevenson M.S., Yuan C.M., Richards K., Delbrook C., Kreitman R.J. et al. (2015) Characterization of CD22 expression in acute lymphoblastic leukemia. Pediatr. Blood Cancer 62, 964–969 10.1002/pbc.25410 25728039 PMC4405453

[26] Langfelder P. and Horvath S. (2008) WGCNA: an R package for weighted correlation network analysis. BMC Bioinformatics 9, 559 10.1186/1471-2105-9-559 19114008 PMC2631488

[27] Langfelder P. and Horvath S. (2007) Eigengene networks for studying the relationships between co-expression modules. BMC Syst. Biol. 1, 54 10.1186/1752-0509-1-54 18031580 PMC2267703

[28] Yu G., Wang L.G., Han Y. and He Q.Y. (2012) clusterProfiler: an R package for comparing biological themes among gene clusters. OMICS 16, 284–287 10.1089/omi.2011.0118 22455463 PMC3339379

[29] Szklarczyk D., Gable A.L., Lyon D., Junge A., Wyder S., Huerta-Cepas J. et al. (2019) STRING v11: protein-protein association networks with increased coverage, supporting functional discovery in genome-wide experimental datasets. Nucleic Acids Res. 47, D607–D613 10.1093/nar/gky1131 30476243 PMC6323986

[30] Chin C.H., Chen S.H., Wu H.H., Ho C.W., Ko M.T. and Lin C.Y. (2014) cytoHubba: identifying hub objects and sub-networks from complex interactome. BMC Syst. Biol. 8, S11 10.1186/1752-0509-8-S4-S11 25521941 PMC4290687

[31] Coustan-Smith E., Song G., Clark C., Key L., Liu P., Mehrpooya M. et al. (2011) New markers for minimal residual disease detection in acute lymphoblastic leukemia. Blood 117, 6267–6276 10.1182/blood-2010-12-324004 21487112 PMC3122946

[32] Balaban N.Q., Schwarz U.S., Riveline D., Goichberg P., Tzur G., Sabanay I. et al. (2001) Force and focal adhesion assembly: a close relationship studied using elastic micropatterned substrates. Nat. Cell Biol. 3, 466–472 10.1038/35074532 11331874

[33] Das S.K. (2010) Regional development of uterine decidualization: molecular signaling by Hoxa-10. Mol. Reprod. Dev. 77, 387–396 10.1002/mrd.21133 19921737 PMC4267754

[34] Rahman M.A., Li M., Li P., Wang H., Dey S.K. and Das S.K. (2006) Hoxa-10 deficiency alters region-specific gene expression and perturbs differentiation of natural killer cells during decidualization. Dev. Biol. 290, 105–117 10.1016/j.ydbio.2005.11.016 16337623 PMC4265803

[35] Sauvageau G., Lansdorp P.M., Eaves C.J., Hogge D.E., Dragowska W.H., Reid D.S. et al. (1994) Differential expression of homeobox genes in functionally distinct CD34+ subpopulations of human bone marrow cells. Proc. Natl. Acad. Sci. U.S.A. 91, 12223–12227 10.1073/pnas.91.25.12223 7527557 PMC45409

[36] Bhatlekar S., Fields J.Z. and Boman B.M. (2014) HOX genes and their role in the development of human cancers. J. Mol. Med. (Berl.) 92, 811–823 10.1007/s00109-014-1181-y 24996520

[37] Guenther M.G., Lawton L.N., Rozovskaia T., Frampton G.M., Levine S.S., Volkert T.L. et al. (2008) Aberrant chromatin at genes encoding stem cell regulators in human mixed-lineage leukemia. Genes Dev. 22, 3403–3408 10.1101/gad.1741408 19141473 PMC2607073

[38] Orlovsky K., Kalinkovich A., Rozovskaia T., Shezen E., Itkin T., Alder H. et al. (2011) Down-regulation of homeobox genes MEIS1 and HOXA in MLL-rearranged acute leukemia impairs engraftment and reduces proliferation. Proc. Natl. Acad. Sci. U.S.A. 108, 7956–7961 10.1073/pnas.1103154108 21518888 PMC3093458

[39] Ferrando A.A., Armstrong S.A., Neuberg D.S., Sallan S.E., Silverman L.B., Korsmeyer S.J. et al. (2003) Gene expression signatures in MLL-rearranged T-lineage and B-precursor acute leukemias: dominance of HOX dysregulation. Blood 102, 262–268 10.1182/blood-2002-10-3221 12637319

[40] Najafi M., Farhood B. and Mortezaee K. (2019) Cancer stem cells (CSCs) in cancer progression and therapy. J. Cell. Physiol. 234, 8381–8395 10.1002/jcp.27740 30417375

[41] Marques L. V.C., Noronha E.P., Andrade F.G., Dos Santos-Bueno F.V., Mansur M.B., Terra-Granado E. et al. (2018) CD44 Expression Profile Varies According to Maturational Subtypes and Molecular Profiles of Pediatric T-Cell Lymphoblastic Leukemia. Front. Oncol. 8, 488 10.3389/fonc.2018.00488 30430079 PMC6220090

[42] Hoofd C., Wang X., Lam S., Jenkins C., Wood B., Giambra V. et al. (2016) CD44 promotes chemoresistance in T-ALL by increased drug efflux. Exp. Hematol. 44, 166e117–171e117 10.1016/j.exphem.2015.12.001 26708679

[43] Armstrong S.A., Staunton J.E., Silverman L.B., Pieters R., den Boer M.L., Minden M.D. et al. (2002) MLL translocations specify a distinct gene expression profile that distinguishes a unique leukemia. Nat. Genet. 30, 41–47 10.1038/ng765 11731795

[44] Tsutsumi S., Taketani T., Nishimura K., Ge X., Taki T., Sugita K. et al. (2003) Two distinct gene expression signatures in pediatric acute lymphoblastic leukemia with MLL rearrangements. Cancer Res. 63, 4882–4887 12941810

[45] Hardy R.R. and Hayakawa K. (2001) B cell development pathways. Annu. Rev. Immunol. 19, 595–621 10.1146/annurev.immunol.19.1.595 11244048

[46] Charrad R.S., Li Y., Delpech B., Balitrand N., Clay D., Jasmin C. et al. (1999) Ligation of the CD44 adhesion molecule reverses blockage of differentiation in human acute myeloid leukemia. Nat. Med. 5, 669–676 10.1038/9518 10371506

[47] Gadhoum Z., Leibovitch M.P., Qi J., Dumenil D., Durand L., Leibovitch S. et al. (2004) CD44: a new means to inhibit acute myeloid leukemia cell proliferation via p27Kip1. Blood 103, 1059–1068 10.1182/blood-2003-04-1218 14525786

[48] Morath I., Hartmann T.N. and Orian-Rousseau V. (2016) CD44: More than a mere stem cell marker. Int. J. Biochem. Cell Biol. 81, 166–173 10.1016/j.biocel.2016.09.009 27640754

[49] Jin L., Hope K.J., Zhai Q., Smadja-Joffe F. and Dick J.E. (2006) Targeting of CD44 eradicates human acute myeloid leukemic stem cells. Nat. Med. 12, 1167–1174 10.1038/nm1483 16998484

[50] Toren A., Bielorai B., Jacob-Hirsch J., Fisher T., Kreiser D., Moran O. et al. (2005) CD133-positive hematopoietic stem cell “stemness” genes contain many genes mutated or abnormally expressed in leukemia. Stem Cells 23, 1142–1153 10.1634/stemcells.2004-0317 16140871

[51] Jang J.W., Song Y., Kim S.H., Kim J. and Seo H.R. (2017) Potential mechanisms of CD133 in cancer stem cells. Life Sci. 184, 25–29 10.1016/j.lfs.2017.07.008 28697984

[52] Mak A.B., Nixon A.M. and Moffat J. (2012) The mixed lineage leukemia (MLL) fusion-associated gene AF4 promotes CD133 transcription. Cancer Res. 72, 1929–1934 10.1158/0008-5472.CAN-11-3589 22337994

[53] Li D., Hu Y., Jin Z., Zhai Y., Tan Y., Sun Y. et al. (2018) TanCAR T cells targeting CD19 and CD133 efficiently eliminate MLL leukemic cells. Leukemia 32, 2012–2016 10.1038/s41375-018-0212-z 30046161

[54] Bueno C., Velasco-Hernandez T., Gutierrez-Aguera F., Zanetti S.R., Baroni M.L., Sanchez-Martinez D. et al. (2019) CD133-directed CAR T-cells for MLL leukemia: on-target, off-tumor myeloablative toxicity. Leukemia 33, 2090–2125 10.1038/s41375-019-0418-8 30778134 PMC6756031

[55] Laaf D., Bojarova P., Elling L. and Kren V. (2019) Galectin-Carbohydrate Interactions in Biomedicine and Biotechnology. Trends Biotechnol. 37, 402–415 10.1016/j.tibtech.2018.10.001 30413271

[56] Wdowiak K., Francuz T., Gallego-Colon E., Ruiz-Agamez N., Kubeczko M., Grochola I. et al. (2018) Galectin Targeted Therapy in Oncology: Current Knowledge and Perspectives. Int. J. Mol. Sci. 19, 210 10.3390/ijms19010210 29320431 PMC5796159

[57] Christiansen M.N., Chik J., Lee L., Anugraham M., Abrahams J.L. and Packer N.H. (2014) Cell surface protein glycosylation in cancer. Proteomics 14, 525–546 10.1002/pmic.201300387 24339177

[58] Greville G., McCann A., Rudd P.M. and Saldova R. (2016) Epigenetic regulation of glycosylation and the impact on chemo-resistance in breast and ovarian cancer. Epigenetics 11, 845–857 10.1080/15592294.2016.1241932 27689695 PMC5193495

[59] Juszczynski P., Rodig S.J., Ouyang J., O'Donnell E., Takeyama K., Mlynarski W. et al. (2010) MLL-rearranged B lymphoblastic leukemias selectively express the immunoregulatory carbohydrate-binding protein galectin-1. Clin. Cancer Res. 16, 2122–2130 10.1158/1078-0432.CCR-09-2765 20332322 PMC2920144

[60] Blanchard H., Bum-Erdene K., Bohari M.H. and Yu X. (2016) Galectin-1 inhibitors and their potential therapeutic applications: a patent review. Exp. Opin. Ther. Pat. 26, 537–554 10.1517/13543776.2016.1163338 26950805

[61] Paz H., Joo E.J., Chou C.H., Fei F., Mayo K.H., Abdel-Azim H. et al. (2018) Treatment of B-cell precursor acute lymphoblastic leukemia with the Galectin-1 inhibitor PTX008. J. Exp. Clin. Cancer Res. 37, 67 10.1186/s13046-018-0721-7 29580262 PMC5870532

[62] Thijssen V.L., Heusschen R., Caers J. and Griffioen A.W. (2015) Galectin expression in cancer diagnosis and prognosis: A systematic review. Biochim. Biophys. Acta 1855, 235–247 25819524 10.1016/j.bbcan.2015.03.003

[63] Miyoshi E., Moriwaki K. and Nakagawa T. (2008) Biological function of fucosylation in cancer biology. J. Biochem. 143, 725–729 10.1093/jb/mvn011 18218651

[64] Shan M., Yang D., Dou H. and Zhang L. (2019) Fucosylation in cancer biology and its clinical applications. Prog. Mol. Biol. Transl. Sci. 162, 93–119 10.1016/bs.pmbts.2019.01.002 30905466

[65] Tu Z., Lin Y.N. and Lin C.H. (2013) Development of fucosyltransferase and fucosidase inhibitors. Chem. Soc. Rev. 42, 4459–4475 10.1039/c3cs60056d 23588106

[66] Dube D.H. and Bertozzi C.R. (2005) Glycans in cancer and inflammation–potential for therapeutics and diagnostics. Nat. Rev. Drug Discov. 4, 477–488 10.1038/nrd1751 15931257

[67] Robinson N.E., de Vries T., Davis R.E., Stults C.L., Watson S.R., van den Eijnden D.H. et al. (1994) Expression of fucosylated antigens and alpha 1,3 fucosyltransferases in human leukaemia cell lines. Glycobiology 4, 317–326 10.1093/glycob/4.3.317 7949657

[68] Taniguchi A., Suga R. and Matsumoto K. (2000) Expression and transcriptional regulation of the human alpha1, 3-fucosyltransferase 4 (FUT4) gene in myeloid and colon adenocarcinoma cell lines. Biochem. Biophys. Res. Commun. 273, 370–376 10.1006/bbrc.2000.2929 10873613

[69] Yi L., Hu Q., Zhou J., Liu Z. and Li H. (2019) Alternative splicing of Ikaros regulates the FUT4/Le(X)-alpha5beta1 integrin-FAK axis in acute lymphoblastic leukemia. Biochem. Biophys. Res. Commun. 510, 128–134 10.1016/j.bbrc.2019.01.064 30683310

